# Evaluation of a Micro-Optical Coherence Tomography for the Corneal Endothelium in an Animal Model

**DOI:** 10.1038/srep29769

**Published:** 2016-07-15

**Authors:** Marcus Ang, Aris Konstantopoulos, Gwendoline Goh, Hla M. Htoon, Xinyi Seah, Nyein Chan Lwin, Xinyu Liu, Si Chen, Linbo Liu, Jodhbir S. Mehta

**Affiliations:** 1 Singapore National Eye Centre, Singapore; 2 Singapore Eye Research Institute, Singapore; 3 Department of Ophthalmology and Visual Science, Duke-NUS Graduate Medical School, Singapore; 4 School of Electrical & Electronic Engineering and School of Chemical & Biomedical Engineering, Nanyang Technological University, Singapore

## Abstract

Recent developments in optical coherence tomography (OCT) systems for the cornea have limited resolution or acquisition speed. In this study we aim to evaluate the use of a ‘micro-OCT’ (μOCT ~1 μm axial resolution) compared to existing imaging modalities using animal models of corneal endothelial disease. We used established cryoinjury and bullous keratopathy models in Sprague Dawley rats comparing *ex vivo* μOCT imaging in normal and diseased eyes to (1) histology; (2) *in vivo* confocal microscopy (IVCM); and (3) scanning electron microscopy (SEM). Qualitative and quantitative comparisons amongst imaging modalities were performed using mean endothelial cell circularity [(4π × Area)/Perimeter^2^] with coefficient of variation (COV). We found that μOCT imaging was able to delineate endothelial cells (with nuclei), detect inflammatory cells, and corneal layers with histology-like resolution, comparable to existing imaging modalities. The mean endothelial cell circularity score was 0.88 ± 0.03, 0.87 ± 0.04 and 0.88 ± 0.05 (P = 0.216) for the SEM, IVCM and μOCT respectively, with SEM producing homogenous endothelial cell images (COV = 0.028) compared to the IVCM (0.051) and μOCT (0.062). In summary, our preliminary study suggests that the μOCT may be useful for achieving non-contact, histology-like images of the cornea for endothelial cell evaluation, which requires further development for *in vivo* imaging.

Corneal diseases are the second most common cause of vision loss^1^,^2^, with over 180 million people worldwide estimated to be suffering from secondary visual impairment^1^. Corneal transplantation still remains the main method for restoring vision once corneal clarity is affected^3^. Thus, evaluation and imaging of the cornea is important for early diagnosis, to allow for timely intervention and prevention of permanent corneal damage. Recent developments in surgical techniques have enabled surgeons to perform selective replacement of the diseased layer of the cornea – which may lead to improved corneal graft survival and surgical outcomes^3^. In particular, selective replacement of both the endothelial layer^4^, and the stromal layer^5^, may confer advantages such as tectonic stability or a rapid visual recovery, compared to replacing the entire cornea during transplantation. Thus, the role of imaging to delineate corneal layers is becoming increasingly important in the pre-operative, intra-operative and post-operative assessment of patients requiring corneal transplantation.

Current imaging techniques such as confocal microscopy and high-frequency ultrasound have limitations such as a narrow field of view or limited resolution respectively^6^. Optical coherence tomography (OCT) has emerged as a promising technique for high-resolution, cross-sectional and en face imaging of the cornea^7^. Existing commercial anterior segment OCT (AS-OCT) systems obtain cross-sections of the cornea with 5–20 μm axial resolution, at a variety of widths (6–16 mm) and depths (2–6 mm)^7^. However, current available AS-OCT technology is unable to clearly image cells within the cornea and may be affected by factors such as scars, artifacts and light scatter that often reduce image quality^8^,^9^.

Recent developments into broadband light sources have allowed for OCT systems to achieve 1–2 μm spatial resolution, termed micro-OCT or μOCT, in an attempt to achieve cellular level imaging *in vivo*^10^,^11^,^12^. By use of a supercontinuum source, μOCT achieves 2 μm × 2 μm × 1 μm resolution (in tissue) at 8 frames per second^13^,^14^. This first μOCT system was shown to provide visualization of many key cellular and sub-cellular features associated with coronary artery diseases^14^, and pulmonary airway diseases *ex vivo*^13^. However, the light source contains pulsed radiations in the visible spectrum (650 nm–700 nm) which is subject to stricter safety constrains in input power. More recently, we have developed a μOCT system using NIR superluminescent diode arrays (SLDs) which make it more suitable for ocular imaging *in vivo* imaging^15^. We had previously described the visualization of corneal endothelial cells using a spectral estimation OCT, which has a 4.7 times better axial resolution compared to spectral domain OCT^16^. However, the image processing speed was too slow for translation to clinical use.

Therefore, in this study we evaluated the SLD array based μOCT system for ‘micro’ (~1 μm) axial resolution, specifically to image the cornea. To the best of our knowledge, this is the first time that three dimensional visualization of corneal endothelium by OCT is validated against gold standard methods. The μOCT imaging system has a spectral bandwidth of 350 nm centered at 930 nm, in order to achieve the best possible axial resolution using near infared (NIR) light. In order to assess the ability of the μOCT system to adequately image the microstructures of the cornea, and in particular, endothelial cells - we used established models to assess normal and damaged cornea. In this preliminary animal study, we compared the μOCT imaging to histology images; and existing imaging modalities such as *in vivo* confocal microscopy (IVCM) and scanning electron microscopy (SEM).

## Materials and Methods

In this study, we used 12 Sprague Dawley rats (aged 8–10 weeks) bred and maintained at the SingHealth Experimental Medical Centre (Singapore General Hospital, Singapore). We utilized two established techniques to induce corneal endothelial injury in one eye of each rat, with the fellow eye serving as the control. First, we used a cryoinjury model previously described^17^, with a cryoprobe made of stainless steel (2.5 mm in diameter; flat tip; ERBE Elektromedizin GmbH, Tübingen, Germany), precooled to −80 °C and gently placed on the central cornea of the rat eye (3 eyes). The cryoprobe was kept on the corneal surface until an ice ball covered the entire corneal surface (approximately 3 seconds duration)^17^. Immediately after freezing, the cryoprobe was freed from the corneal surface with irrigation with a balanced salt solution, and the cornea was allowed to thaw spontaneously. The second model we used to induce bullous keratopathy^18^, was performed with benzalkonium chloride (BAK) 0.05%, which was injected into the anterior chamber of the rat eyes (3 eyes). Briefly, the anterior chamber of one eye was punctured using a 30 G needle under anesthesia and rinsed with BAK for 90 seconds, followed by rinsing with 0.9% sodium chloride for another 90 seconds. The corneal puncture was sealed with a small air bubble. Antibiotic ointment was applied to the eyes at the end of each procedure. Our study was conducted with approval from the Institutional Animal Care and Use Committee of Singapore Health Services; and all animals were treated according to tenets of the Association for Research in Vision and Ophthalmology’s statement for the Use of Animals in Ophthalmic and Vision Research.

### Micro-Optical Coherence Tomography (μOCT)

Optical coherence tomography measures the electric field amplitude of light that is elastically scattered from within tissue in three dimensions. Depth or axial (z) ranging is achieved by interferometric measurement of the optical delay of light returned from the sample. The μOCT system we have described here is a spectral-domain OCT, implemented with several key improvements to standard OCT that yields high resolution in both lateral and axial directions as previously described^15^. In brief, the combined output of two SLD arrays (Superlum Broadlighters T-850-HP and Exalos Ultra-Broadband EBS4C32) provides the high-bandwidth (755–1105 nm), short coherence length light necessary for high axial resolution of 1.3 μm in air. A typical OCT system includes an interferometer with the reference and sample arms intersecting at a beamsplitter. The device is equipped with a 10X objective lens and a 20X objective lens, which provides a lateral resolution of 2.5 μm and 1.3 μm respectively. The effective beam diameter at the the back aperture of the objective lens was 2.6 mm (1% power level) so that it was under-filled. A telecentric scanning configureation was assumed to perform a sectional scan across a transverse range of 0.872 mm by 0.872 mm. The total power incident on the sample was less than 2 mW. Custom software was employed to control the galvanometer scanning motors while acquiring spectral data from the two-line scan cameras. In order to detect the spectral interference signal across the entire illumination bandwidth, we employed two spectrometers based on an InGaAs camera (Sensors Unlimited GL2048L) and a Si camera (E2V, AViiVA EM4) respectively. The system operates with a user-configurable line and frame rates and customizable scan geometry; typical settings are 60 frames per second, 1024 A-lines per frame in a linear scan, and 0.872 mm by 0.872 mm (X by Z) for a cross-sectional image. The transverse scanning step size was 0.85 μm is less than half of the μOCT beam spot size (2.5 μm) to satisfy the requirement set by Nyquist sampling therom. A three dimensional image could be formed by acquiring a time-series stack of 1024 B-mode (cross-sectional) μOCT images within 17 seconds.

### Anterior segment evaluation and histology

Preoperatively and 3 days after the interventions, examinations including AS-OCT (RTvue, Optovue, Fremont, CA), *in vivo* confocal microscopy i.e. IVCM (HRT3 Rostock module; Heidelberg Engineering GmbH, Heidelberg, Germany) and slit-lamp photography (FS-3V Zoom Photo Slit Lamp, Nikon, Tokyo, Japan) were performed. All animals were then sacrificed and *ex vivo* imaging was performed with the μOCT system, before flat mount preparations of treated and untreated corneas to evaluate endothelial cells and cross-sectional histology. Corneas were fixed in 4% paraformaldehyde, dehydrated and embedded in paraffin blocks for sectioning at 5 μm for haematoxylin and eosin (H&E) staining as previously described^19^. In brief, sections were immersed in hematoxylin (Sigma Aldrich, St. Louis, MO, USA) for 2 minutes and counter stained with eosin (Sigma Aldrich, St. Louis, MO, USA) solutions for another 2 minutes before soaking with pure xylene to remove traces of ethanol, dried and imaged using a light microscope (Nikon C2 confocal microscope). The corneal buttons excised from the enucleated eyes were placed endothelial side up and stained with alizarin red S (0.50%; pH 4.2) for 3 minutes, and then were washed in wash buffer solution twice for 2 minutes. The corneas were then mounted on a glass slide endothelial side up under a cover slip, and were imaged using an inverted light microscope (Nikon C2 Confocal microscope).

### Scanning electron microscopy (SEM)

We also evaluated the changes in the corneal endothelium by performing SEM in both the diseased eyes and control eyes. The globes were immersed in a fixative solution, containing 2.5% glutaraldehyde in 0.1 M sodium cacodylate (pH 7.4; Electron Microscopy Sciences, Hatfield, PA) overnight at 4 °C. The corneas were excised from the globes, washed three times in distilled water for 10 min each, and were kept in 1% osmium tetroxide (FMB, Singapore) at 22 °C for 2 h for final fixation. The corneas were then dehydrated through serial dilutions of ethanol (25%, 50%, 75%, 95%, and 100% each for 10 min, with the 100% twice). The samples were then dried in a critical point dryer (BALTEC, Balzers, Liechtenstein) and mounted on SEM stubs using carbon adhesive tabs. Samples were then sputter-coated with a 10 nm thick layer of gold (BALTEC) and examined with a scanning electron microscope (JSM-5600; JEOL, Tokyo, Japan).

### Statistical Analysis

All numeric data obtained were expressed as mean ± standard deviation. Comparisons of mean endothelial cell circularity with coefficient of variation (COV) were statistically analyzed using two-way ANOVA followed by post-hoc Bonferroni test for multiple comparisons. Two masked assessors obtained morphometric data of the area and perimeter of fifty randomly selected cells from scans of each imaging technique (μOCT, IVCM and SEM) were manually outlined by point-to-point tracing of the cell borders using the National Institutes of Health Image J 1.38X (NIH, Bethesda, MD) software. Cell circularity was then determined using the formula:


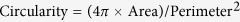


where a value approaching 1.0 indicated a circular profile^20^. Hence, normal hexagonal endothelial cells will have a profile closer to 1.0 as opposed to damaged endothelial cells. Alpha was set at a significance level of 5%. All analyses were performed using STATA version 11 (StataCorp LP, College Station, Texas, USA).

## Results

**Figure 1:**
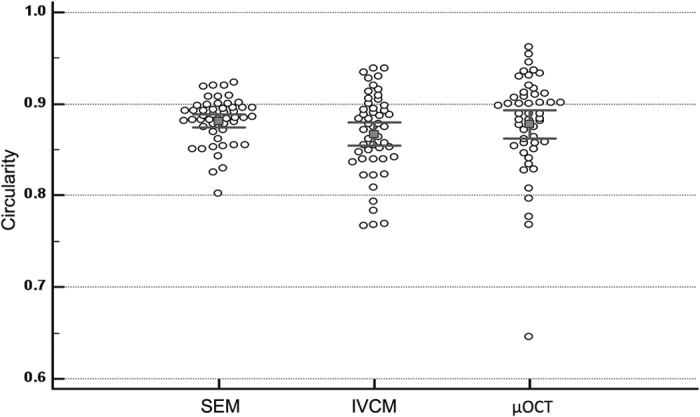
Endothelial cell circularity score i.e. (4π × Area)/Perimeter^2^ where a value approaching 1.0 indicated a more circular and hexagonal profile, compared amongst the three imaging techniques using scanning electron microscopy (SEM), *vivo* confocal microscopy (IVCM), and micro-optical coherence tomography (μOCT); P = 0.216.

**Figure 2:**
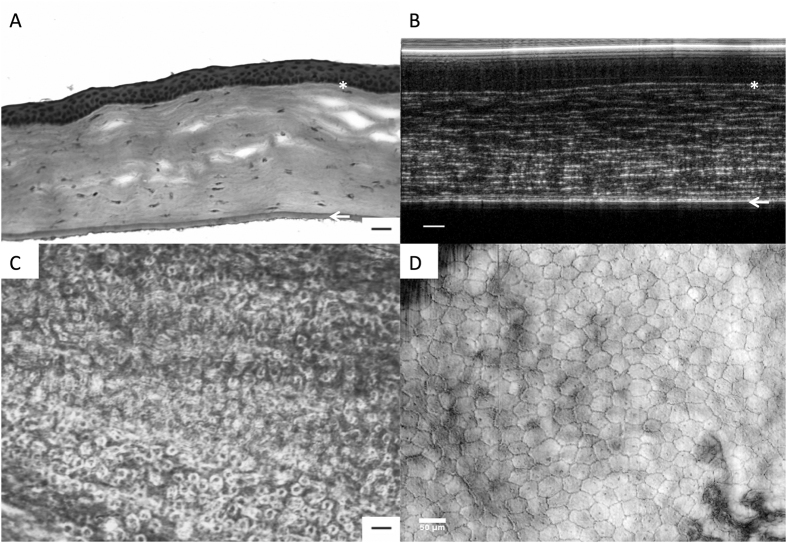
Histology images compared to μOCT scan images of the normal mouse cornea. (**A**) Cross-sectional histology sections using H&E stain (20x magnification, scale bar = 20 μm). *Indicates Bowman’s layer. Epithelium lies above the Bowman’s layer. Arrow indicates Descemet membrane, corneal stromal layer lies in between. Endothelial cell layer can be seen just below the Descemet membrane. (**B**) Cross-sectional B-scan μOCT images were able to delineate the corresponsing distinct layers of the cornea with similar resolution to histology images i.e. *Bowman’s layer, collagen fibrils in stroma, Descemet membrane (arrow) and endothelial cell layer below (scale bar = 40 μm). Epithelium lies above the Bowman’s layer. Arrow indicates Descemet membrane, corneal stromal layer lies in between. Endothelial cell layer can be seen just below the Descemet membrane. (**C**) Endothelial cell layer histology image using Alizarin S Red (20x magnification, scale bar = 20 μm). (**D**) En face μOCT scan of the normal endothelial cells without artifacts from histology fixing and staining (scale bar = 40 μm) was able to detect the presence of nuclei and villi.

We found that the circularity scores were comparable between all 3 imaging modalities in the normal endothelial cell analysis of control eyes (n = 12). The mean circularity score was 0.88 ± 0.03, 0.87 ± 0.04 and 0.88 ± 0.05 (P = 0.216) for the SEM, IVCM and μOCT image analysis respectively – Fig. 1. Each imaging modality was able to outline the normal endothelial cells, with the SEM producing homogenous endothelial cell images (COV = 0.028) compared to the IVCM (0.051) and μOCT (0.062). When compared to histology images, the en face μOCT imaging were able to delineate the endothelial cells clearly without artifacts from fixing techniques; while B-scan μOCT images successfully demonstrated distinct layers of normal cornea i.e. epithelium, Bowman’s layer, stromal layers and Descemet’s membrane (DM). The high-resolution μOCT was also able to delineate the endothelial cell nuclei, which are not usually visible when imaged by IVCM or specular microscopy – Fig. 2.

**Figure 3:**
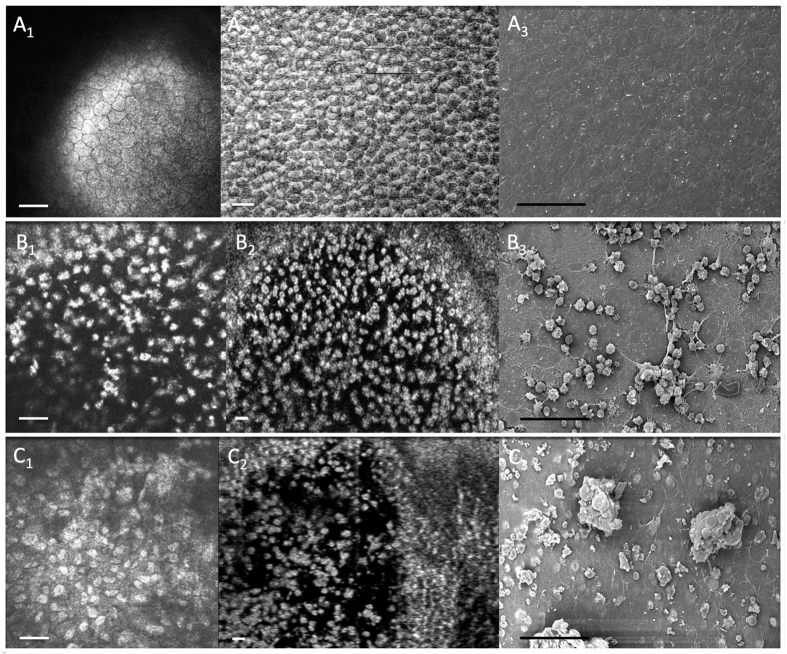
Endothelial cell imaging using *in vivo* confocal microscopy (left), micro-optical coherence tomography (center) and scanning electron microscopy (right) in control eyes (**A**), cryoinjury model eyes (**B**) and benzakolnium chloride injury model (**C**). A_1_, B_1_ and C_1_: En-face images of rats cornea pre (A_1_) and post-injury (B_1_, C_1_) using *In vivo* confocal Microscopy (scale bar = 50 μm). A_2_, B_2_ and C_2_: En-face images of rats cornea pre (A_2_) and post-injury (B_2_,C_2_) using micro-OCT (Scale bar = 40 μm). A_3_, B_3_, and C_3_: Cross sectional images of rats cornea Pre (A_3_) and post-injury (B_3_,C_3_) using Scanning Electron Microscopy (2000X magnification) (scale bar = 50 μm).

**Figure 4:**
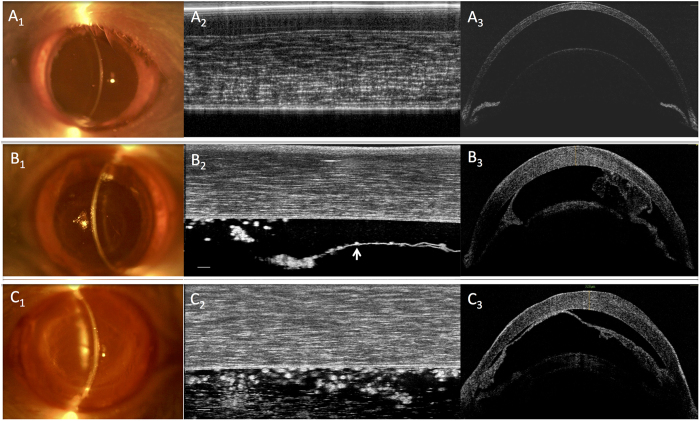
Slit lamp follow up and Cross sectional images of (**A**) normal rat cornea (**B**) Cryo-injury rat cornea (**C**) Chemical (BAK) injury rat cornea. A_1_, B_1_ and C_1_: Slit lamp examination of rats cornea Pre (A_1_) and post-injury (B_1_,C_1_) (30X magnification). A_2_, B_2_ and C_2_: Cross sectional images of rats cornea Pre (A_2_) and post-injury (B_2_,C_2_) using micro-OCT (Scale bar = 40 μm). A_3_, B_3_, and C_3_: Cross sectional images of rats cornea Pre (A_3_) and post-injury (B_3_,C_3_) using Optovue OCT.

We also found that the μOCT was able to image the endothelial cell surface in the central cornea in all the samples, using the coronal reconstruction or en face view of the serial scans in the cryoinjury (n = 6) and BAK injury (n = 6) eyes. The μOCT images reflected the loss of hexagonality, disruption of the endothelial cell layer and signs of inflammation in the same central cornea, which was also detected in the IVCM and SEM images – representative examples in Fig. 3. After 3 days from endothelial injury, we observed an increase in central corneal thickness in the cryoinjury model (n = 6, mean ± standard deviation: 320 ± 60 μm, P = 0.047) and BAK injury model (n = 6, 371 ± 90 μm, P = 0.004) compared to the control eyes (n = 12, 169 ± 10 μm) as measured by AS-OCT, which is optimized and validated for measuring corneal thickness. While the conventional AS-OCT was able to detect the gross changes in the cornea, we found that the μOCT was better able to delineate the layers of the cornea in the control eyes, especially the epithelium, Bowman’s layer, and Descemet membrane; as well as that seen in the injury model eyes – Fig. 4. The μOCT also detected subtle changes in the stromal layer where the cryoinjury eyes had anterior stromal scarring with cells seen on the damaged DM; while the BAK injured eyes had more edema with minimal stromal haze and more inflammatory cells on the DM surface.

## Discussion

In this preliminary study, we describe the use of a new μOCT system that utilizes a very broad bandwidth light source and common-path spectral-domain OCT (SD-OCT) technology to provide 1-μm-axial resolution (in tissue) scans of the cornea. We found that the novel μOCT system was able to produce ‘histology-resolution’ images using both the cross-sectional views of the cornea, as well as en face views of the endothelial surface – without suffering from the artifacts usually introduced by histology fixing techniques. With this micro-resolution of 1-μm, accurate measurements of the corneal layers, as well as finer detail of structures such as inflammatory cells within the stroma; or epithelial and DM damage, may be obtained directly from a time-series stack of B-mode (cross-sectional) μOCT images – potentially a significant improvement over current commercial ASOCT imaging. Moreover, coronal reconstruction from rapid serial μOCT scans also allowed rapid non-contact imaging of the endothelial cell layer compared to the ‘contact’ IVCM and time consuming *in vitro* SEM – but with a much larger field of view (IVCM: 400 × 400 μm; SEM: 200 × 1300 μm). We found that the μOCT produced endothelial cell imaging with homogenous circularity scores as a surrogate for hexagonality, as we recognize that direct comparisons using endothelial cell density was not possible due to the artifacts from histology fixing and the lack of a reference across imaging modalities. The high-resolution μOCT was able to delineate the endothelial cell nuclei, which are not usually visible when imaged by IVCM or specular microscopy, which may have potential clinical applications such as detection of early endothelial damage, as we continue to develop the μOCT for *in vivo* use^21^.

Since its first *in vivo* use for the retina, OCT imaging has revolutionized our ability to evaluate the eye and its structures on a microscopic level^22^. Currently, commercially available ultrahigh-resolution OCT may provide a potential improvement in performance, enabling imaging of corneal cells or even delineation of micro-vascular structures, which had previously only been possible with IVCM or histopathology^23^,^24^. In combination with image processing and segmentation techniques, we describe a further improved μOCT that permits the quantitative measurement of corneal microanatomy and morphology, i.e. non-contact visualization of endothelial cells using en face OCT reconstruction^25^. Previous studies have examined the correlation of conventional resolution OCT and histology of the retina in animals, but found discrepancies due to image resolution and histology fixation changes^26^. Here, we compared our μOCT images with SEM and IVCM to show that μOCT provided rapid, non-contact *ex vivo* histology-like images for the cornea and endothelium. Moreover, while previous prototype high-resolution OCT systems were able to visualize corneal layers^27^, the system described here was able to produce images with a similar axial resolution and field of view, but with additional cellular detail such as the presence of inflammatory cells within the stromal and endothelial layer. The potential clinical applications of *in vivo* imaging of the corneal endothelial cells include monitoring corneal endothelial cell count and morphology to guide surgery^28^, improving corneal endothelial cell imaging to compare endothelial keratoplasty techniques^29^, or even early detection of post-keratoplasty rejection by looking at inflammatory cells to differentiate rejection from infection^30^.

The advantages of OCT are well known^31^, with image resolution improving over time to achieve histology-like images; and non-contact *in vivo* images obtained in real time that potentially allows surgical guidance and functional imaging^32^. However, the disadvantages of OCT, especially μOCT, include the trade-off between lateral resolution and depth of focus limited axial imaging range, and imaging speed^11^. The maximum depth of focus of μOCT used in this study is limited by confocal parameter to approximately 150 μm, which can be mitigated by use of depth of focus extension techniques^33^,^34^,^35^. Likewise, the axial imaging range (ranging depth) was 0.5 mm, which could be solved by use of a line scan camera of larger pixel numbers and/or full-range OCT imaging^36^. While IVCM generally achieves an axial resolution of 4–10 μm and a transverse resolution of 2–6 μm, while a previously described full-field OCT offered a axial and transverse resolution of 2–3 μm, image acquisition time was relatively lengthy (1.5 s/image), requiring the samples to be completely immobile^37^. Full-field optical coherence microscopy (FF-OCM) has also been used to visualize endothelial cells, but requires acquisition times of ~20–100 times longer than that of spectral domain OCT, which makes it difficult to translate it for clinical use^27^. In the current system, we achieved 1 μm axial resolution with a higher image acquisition speed of 60 kHz A-line. Since high-speed imaging is important to reduce motion artifact and enable clinical imaging applications, the future development of μOCT will be focused on improving image acquisition speed and motion tracking. One promising solution to the speed issue may be to develop a swept source μOCT system which can achieve an A-line speed up to 4 MHz^38^.

We recognize that our results are from a pilot study in a small number of eyes where this novel technology was tested in *ex vivo* rat eyes. Ideally, a larger number of eyes with *in vivo* analysis of corneal endothelial cell parameters such as that from specular microscopy could have been performed, but we used an *in vivo* animal model to evaluate both normal and damaged endothelial cells, where such parameters are not applicable. Nonetheless, we provide promising results from this preliminary study that used a novel μOCT system to provide rapid non-contact en face views of the corneal endothelium, with comparative repeatability compared to other conventional imaging techniques. The ability to delineate the endothelial nuclei and inflammatory cells have potential clinical applications, and future developments in image processing will improve image resolution and the depth of penetration will also allow us to perform *in vivo* imaging in human corneas. In summary, we described a novel μOCT system using a high-resolution spectral domain OCT imaging technique with established methods and algorithms, to potentially achieve histology-like images within the rat cornea. This preliminary study suggests that the μOCT system is able to obtain endothelial cell imaging with adequate resolution compared to histology and other existing techniques. Further *in vivo* studies, and ultimately, translation for human use *in vivo* would be required to establish this promising μOCT system for clinical applications.

## Additional Information

**How to cite this article**: Ang, M. *et al*. Evaluation of a Micro-Optical Coherence Tomography for the Corneal Endothelium in an Animal Model. *Sci. Rep.* **6**, 29769; doi: 10.1038/srep29769 (2016).

## References

[b1] Whitcher J. P., Srinivasan M. & Upadhyay M. P. Corneal blindness: a global perspective. Bull World Health Organ 79, 214–221 (2001). 11285665 PMC2566379

[b2] Foster A. & Resnikoff S. The impact of Vision 2020 on global blindness. Eye (Lond) 19, 1133–1135 (2005). 16304595 10.1038/sj.eye.6701973

[b3] Tan D. T., Dart J. K., Holland E. J. & Kinoshita S. Corneal transplantation. Lancet 379, 1749–1761 (2012). 22559901 10.1016/S0140-6736(12)60437-1

[b4] Ang M. . Endothelial cell loss and graft survival after Descemet’s stripping automated endothelial keratoplasty and penetrating keratoplasty. Ophthalmology 119, 2239–2244 (2012). 22885122 10.1016/j.ophtha.2012.06.012

[b5] Han D. C., Mehta J. S., Por Y. M., Htoon H. M. & Tan D. T. Comparison of outcomes of lamellar keratoplasty and penetrating keratoplasty in keratoconus. Am J Ophthalmol 148, 744–751 e741 (2009). 10.1016/j.ajo.2009.05.028 19589495

[b6] Rio-Cristobal A. & Martin R. Corneal assessment technologies: current status. Surv Ophthalmol 59, 599–614 (2014). 25223496 10.1016/j.survophthal.2014.05.001

[b7] Ang M. . Anterior segment optical coherence tomography study of the cornea and anterior segment in adult ethnic South Asian Indian eyes. Invest Ophthalmol Vis Sci. 53, 120–125 (2012). 22025574 10.1167/iovs.11-8386

[b8] Girard M. J., Strouthidis N. G., Ethier C. R. & Mari J. M. Shadow removal and contrast enhancement in optical coherence tomography images of the human optic nerve head. Invest Ophthalmol Vis Sci. 52, 7738–7748 (2011). 21551412 10.1167/iovs.10-6925

[b9] Mari J. M., Strouthidis N. G., Park S. C. & Girard M. J. Enhancement of lamina cribrosa visibility in optical coherence tomography images using adaptive compensation. Invest Ophthalmol Vis Sci. 54, 2238–2247 (2013). 23449723 10.1167/iovs.12-11327

[b10] Liu L. . Method for quantitative study of airway functional microanatomy using micro-optical coherence tomography. PloS one 8, e54473 (2013). 23372732 10.1371/journal.pone.0054473 PMC3553101

[b11] Liu L. . Imaging the subcellular structure of human coronary atherosclerosis using micro-optical coherence tomography. Nature Medicine 17, 1010–1014 (2011). 10.1038/nm.2409 PMC3151347 21743452

[b12] Liu L. . An autoregulatory mechanism governing mucociliary transport is sensitive to mucus load. American journal of respiratory cell and molecular biology 51, 485–493 (2014). 24937762 10.1165/rcmb.2013-0499MA PMC4189485

[b13] Liu L. . Method for Quantitative Study of Airway Functional Microanatomy Using Micro-Optical Coherence Tomography. Plos One 8, e54473 (2013). 23372732 10.1371/journal.pone.0054473 PMC3553101

[b14] Liu L. . Imaging the subcellular structure of human coronary atherosclerosis using micro-optical coherence tomography. Nat Med. 17, 1010–1014 (2011). 21743452 10.1038/nm.2409 PMC3151347

[b15] Cui D., Liu X. & Zhang J. . Dual spectrometer system with spectral compounding for 1-µm optical coherence tomography *in vivo*. Opt. Lett. 39(23), 6727–30 (2014). 25490663 10.1364/OL.39.006727

[b16] Liu X., Chen S., Cui D., Yu X. & Liu L. Spectral estimation optical coherence tomography for axial super-resolution. Optics Express 23, 26521–26532 (2015). 26480165 10.1364/OE.23.026521

[b17] Han S. B. . A mouse model of corneal endothelial decompensation using cryoinjury. Mol Vis 19, 1222–1230 (2013). 23761724 PMC3675054

[b18] Bredow L., Schwartzkopff J. & Reinhard T. Regeneration of corneal endothelial cells following keratoplasty in rats with bullous keratopathy. Mol Vis. 20, 683–690 (2014). 24883013 PMC4037534

[b19] Han S. B. . Mice with a Targeted Disruption of Slc4a11 Model the Progressive Corneal Changes of Congenital Hereditary Endothelial Dystrophy. Invest Ophthalmol Vis Sci. (2013). 10.1167/iovs.13-12089 23942972

[b20] Peh G. S. . Optimization of human corneal endothelial cell culture: density dependency of successful cultures *in vitro*. BMC Res Notes 6, 176 (2013). 23641909 10.1186/1756-0500-6-176 PMC3659058

[b21] Patel D. V., Phua Y. S. & McGhee C. N. Clinical and microstructural analysis of patients with hyper-reflective corneal endothelial nuclei imaged by *in vivo* confocal microscopy. Exp Eye Res. 82, 682–687 (2006). 16359661 10.1016/j.exer.2005.09.006

[b22] Swanson E. A. . *In vivo* retinal imaging by optical coherence tomography. Opt Lett 18, 1864–1866 (1993). 19829430 10.1364/ol.18.001864

[b23] Ang M. . Optical Coherence Tomography Angiography for Anterior Segment Vasculature Imaging. Ophthalmology 122, 1740–1747 (2015). 26088621 10.1016/j.ophtha.2015.05.017

[b24] Ang M., Cai Y., Shahipasand S. . En face optical coherence tomography angiography for corneal neovascularisation. Br J Ophthalmol 100(5), 616–21 (2016). 26311064 10.1136/bjophthalmol-2015-307338

[b25] Girard M. J. . Enhancement of Corneal Visibility in Optical Coherence Tomography Images Using Corneal Adaptive Compensation. Transl Vis Sci Technol 4, 3 (2015). 26046005 10.1167/tvst.4.3.3 PMC4451879

[b26] Drexler W. . Ultrahigh-resolution ophthalmic optical coherence tomography. Nat Med. 7, 502–507 (2001). 11283681 10.1038/86589 PMC1950821

[b27] Christopoulos V. . *In vivo* corneal high-speed, ultra high-resolution optical coherence tomography. Arch Ophthalmol 125, 1027–1035 (2007). 17698748 10.1001/archopht.125.8.1027 PMC2136433

[b28] Ang M. . Endothelial keratoplasty after failed penetrating keratoplasty: an alternative to repeat penetrating keratoplasty. Am J Ophthalmol 158, 1221-1227 e1221 (2014). 10.1016/j.ajo.2014.08.024 25152499

[b29] Ang M., Wilkins M. R., Mehta J. S. & Tan D. Descemet membrane endothelial keratoplasty. Br J Ophthalmol 100, 15–21 (2016). 25990654 10.1136/bjophthalmol-2015-306837

[b30] Ang M., Sng C. C., Chee S. P., Tan D. T. & Mehta J. S. Outcomes of corneal transplantation for irreversible corneal decompensation secondary to corneal endotheliitis in Asian eyes. Am J Ophthalmol 156, 260–266 e262 (2013). 10.1016/j.ajo.2013.03.020 23622566

[b31] Fujimoto J. G. Optical coherence tomography for ultrahigh resolution *in vivo* imaging. Nature Biotechnology 21, 1361–1367 (2003). 10.1038/nbt892 14595364

[b32] Jia Y. . Split-spectrum amplitude-decorrelation angiography with optical coherence tomography. Optics Express 20, 4710–4725 (2012). 22418228 10.1364/OE.20.004710 PMC3381646

[b33] Leitgeb R. A., Villiger M., Bachmann A. H., Steinmann L. & Lasser T. Extended focus depth for Fourier domain optical coherence microscopy. Opt. Lett. 31, 2450–2452 (2006). 16880852 10.1364/ol.31.002450

[b34] Yu X. . Depth extension and sidelobe suppression in optical coherence tomography using pupil filters. Optics Express 22, 26956–26966 (2014). 25401845 10.1364/OE.22.026956

[b35] Liu L., Liu C., Howe W. C., Sheppard C. J. R. & Chen N. Binary-phase spatial filter for real-time swept-source optical coherence microscopy. Optics Letters 32, 2375–2377 (2007). 17700790 10.1364/ol.32.002375

[b36] Sarunic M., Choma M. A., Yang C. & Izatt J. A. Instantaneous complex conjugate resolved spectral domain and swept-source OCT using 3x3 fiber couplers. Optics Express 13, 957–967 (2005). 19494959 10.1364/opex.13.000957

[b37] Akiba M. . Ultrahigh-resolution imaging of human donor cornea using full-field optical coherence tomography. J Biomed Opt. 12, 041202 (2007). 17867791 10.1117/1.2764461

[b38] Fechtig D. J., Schmoll T., Grajciar B. . Line-field parallel swept source interferometric imaging at up to 1 MHz. Opt. Lett. 39(18), 5333–6 (2014). 26466264 10.1364/OL.39.005333

